# Investigating the relationship between social media use and the attitudes towards nutrition and body image shame among Iranian female students: A cross-sectional study

**DOI:** 10.1097/MD.0000000000041383

**Published:** 2025-01-24

**Authors:** Sogol Keyvanpour, Masoumeh Namazi, Fatemeh Hosseini, Narges Ebrahimi

**Affiliations:** a Students Scientific Research Center, Tehran University of Medical Sciences, Tehran, Iran; b Department of Midwifery and Reproductive Health, School of Nursing and Midwifery, Tehran University of Medical Sciences, Tehran, Iran; c Department of Epidemiology and Biostatistics, School of Public Health, Tehran University of Medical Sciences, Tehran, Iran; d Department of Health Education and Promotion, School of Public Health, Tehran University of Medical Sciences, Tehran, Iran.

**Keywords:** body image, body mass index, body shame, confirmatory model, nutrition, social media

## Abstract

Social media are Internet-based services that allow participation in online communities and exchanges. Considering the high and increasing statistics of the use of social media all over the world and its impact on people’s lives, the present study aimed to determine the relationship between social media and nutritional attitudes and body image shame among Iranian female students. This cross-sectional study was performed on 201 female student of Tehran University of Medical Sciences in Tehran, Iran from May to December 2023. Data collection was done using a paper form. Data collection tools included a questionnaire on demographic information, Jahanbani social media use questionnaire, nutrition attitude questionnaire (EAT-26), and Body Image Shame Questionnaire (BISS). SPSS software version 28 was used for the descriptive analysis of the data, to examine the relationships between the studied variables, and to measure and evaluate the accuracy of the AMOS model. Based on the reported results, the indicator of social media usage had a significant and positive direct effect on the attitude towards nutrition of students (*P* < .05). Additionally, social media usage had a significant and positive direct effect on the indicator of body image shame of students (*P* < .05). Furthermore, students’ attitude towards nutrition had a significant and positive effect on the indicator of body image shame of students (*P* < .001). The findings underscore the need for interventions that address the negative effects of social media on young women’s perceptions of their bodies and their relationship with food. By promoting media literacy, body positivity, and healthy nutritional attitudes, it is possible to mitigate the harmful effects of social media and support the well-being of young women.

## 1. Introduction

Social media has swiftly become a pervasive force, influencing various aspects of individuals’ lives in both positive and negative ways. On the positive side, it offers access to real-time information, fosters business opportunities, facilitates knowledge sharing, and enhances social interaction and communication.^[1]^ For children and adolescents, social media can boost awareness, improve social skills, and inspire motivation. However, these advantages are accompanied by significant downsides.^[1–3]^ Excessive use of social media can lead to time wastage, digital addiction, insufficient rest, and susceptibility to hacking. Among young people, specific issues include distorted body image, promotion of risky behaviors, academic struggles, and declining academic performance.^[3]^ Moreover, social media has been associated with unhealthy nutritional attitudes, particularly among women aged 20 to 29. The portrayal of idealized body types on these platforms often intensifies body dissatisfaction, increasing the desire for thinness and the risk of eating disorders.^[4]^

Proper nutrition is essential for human development, as poor dietary habits can compromise physical health and increase vulnerability to cognitive and behavioral issues. Nutritional attitudes – defined as beliefs, emotions, and behaviors related to food – are influenced by factors such as age, gender, and psychological stress. Research indicates that incorrect nutritional attitudes become more common with age and are more prevalent in women.^[5]^ Individuals with shorter stature or higher BMI are also more likely to develop unhealthy nutritional attitudes, often using them as coping mechanisms for psychological stress.^[5,6]^ Traits like impulsivity and stubbornness are linked to restrictive eating habits and obsessive thoughts about food, which heighten the risks of obesity and disordered eating.^[7,8]^

Recent statistics reveal that about 95% of eating disorder cases occur in individuals aged 12 to 25, with 1 death occurring every hour due to complications from these disorders.^[9]^ People with abnormal nutritional attitudes are also more likely to experience depressive symptoms.^[10]^ Research highlights social media’s role in influencing nutritional attitudes by amplifying body dissatisfaction. By promoting thinness as an ideal, social media fosters weight loss motivations and body dissatisfaction, leading to body shame.^[11]^ Body shame, characterized by self-blame and critical perceptions of one’s appearance, often stems from dissatisfaction with one’s physical appearance, encompassing various sensory perceptions like touch or smell. Studies show that individuals struggling with body dissatisfaction are at a higher risk of adopting unhealthy nutritional attitudes, using these as a response to feelings of inadequacy.^[12]^

Body shaming involves criticism, ridicule, or self-blame related to physical appearance. It can originate from external sources, such as social media, or internalized self-criticism. This includes mockery of weight, height, hair, body shape, muscle mass, or facial features. Such criticism has significant negative effects on psychological well-being, with studies linking social media dependence to unhealthy nutritional attitudes and increased body dissatisfaction.^[13]^ Time spent on social media is associated with heightened body dissatisfaction, regardless of the platform type.^[14]^ Given the widespread use of social media globally and within Iran, understanding its effects on users’ lifestyles, nutritional attitudes, and body perceptions is critical. Prior research has examined the adverse effects of social media on body image and eating behaviors.^[15]^

This study adopts social comparison theory, which suggests that individuals evaluate themselves based on comparisons with others, particularly when exposed to idealized online images. Frequent exposure to curated body ideals on social media can lead female students to make unfavorable comparisons, exacerbating body dissatisfaction and fostering unhealthy nutritional attitudes.^[16]^ Grounded in this framework, the study aims to explore the relationship between social media usage, nutritional attitudes, and body image shame among Iranian female students. The hypothesis posits that higher social media use correlates with a greater likelihood of incorrect nutritional attitudes and intensified body image shame. Additionally, it suggests that incorrect nutritional attitudes mediate the link between social media use and body image shame, offering a deeper understanding of social media’s impact on health-related behaviors within this cultural context.

## 2. Study objectives

1. Assessment of Nutritional Attitudes Among Female Students at Tehran University of Medical Sciences
2. Evaluation of Body Image Shame Among Female Students at Tehran University of Medical Sciences
3. Examination of the Impact of Social Media on Nutritional Attitudes Among Female Students at Tehran University of Medical Sciences
4. Analysis of the Impact of Social Media on Body Image Shame Among Female Students at Tehran University of Medical Sciences

## 3. Research hypotheses

1. There is a relationship between social media usage and nutritional attitudes among female students at Tehran University of Medical Sciences.
2. There is a relationship between social media usage and body image shame among female students at Tehran University of Medical Sciences.

## 4. Method and materials

This cross-sectional study was conducted among female students at Tehran University of Medical Sciences in Tehran, Iran, from May to December 2023. Tehran University of Medical Sciences is the largest medical university in Iran, located in the country’s capital, Tehran. The university comprises 10 faculties, including the Faculty of Medicine, Nursing and Midwifery, Pharmacy, Allied Medical Sciences, Advanced Technologies, Nutrition, Rehabilitation, Public Health, Dentistry, and Iranian Medicine. Ethical approval for this study was granted by the Tehran University of Medical Sciences Ethics Committee (approval number: IR.TUMS.MEDICINE.REC.1402.143). Informed consent was obtained from all participants before data collection. The target population was selected using convenience sampling due to logistical accessibility, with participants drawn from various fields, including medicine, dentistry, pharmacy, nursing, midwifery, and allied health. This sampling approach facilitated recruitment but may limit the generalizability of the findings beyond this specific group, a limitation acknowledged in this study. Participants were included if they were currently enrolled as female students at Tehran University of Medical Sciences, willing to participate, and able to complete the questionnaires. Exclusion criteria included incomplete questionnaire responses and unwillingness to participate.

Based on the study of Jianting Shen et al,^[17]^ the mean and SD of the effect of media on negative body image were reported to be 3.21 and 0.73, respectively. Considering the *α* = 0.05, the minimum calculated sample size is 200 people.^[18]^


$$\begin{array}{l} n=\frac{{\left(Z_{1-(a/2)}S\right)}^{2}}{d^{2}} \end{array}$$

Data collection was done using a paper form. The data collection tools included a questionnaire on demographic information, Jahanbani social media use questionnaire, nutrition attitude questionnaire (EAT-26), and Body Image Shame Questionnaire (BISS).

- The demographic information questionnaire consisted of 11 questions included age, marital status, height, weight, body mass index (BMI), physical or mental illness, parents’ education level, parents’ occupation and economic status, degree and field of study.

- Jahanbani social media use questionnaire: It was designed by Jahanbani et al in 2017 and includes 19 questions in a 5-point Likert scale (from very little to very much) and scores from 1 to 5, which include questions in 3 areas of frequency of use (questions 1–5), the type of use (questions 6–12) and the level of users’ trust in social networks (questions 13–19). The lower limit of the questionnaire score is 10 and the upper limit is 95. If the scores of the questionnaire are between 19 and 38, the amount of use of networks and social media is weak, the scores between 38 and 57 indicate the amount of use at an average level, and the scores above 57 indicate the amount of use at the high level. The reliability of the questionnaire was confirmed to be 0.90 using Spearman correlation coefficient and the internal reliability has been confirmed by using Cronbach alpha (0.85).^[19]^

- Nutrition attitude questionnaire (EAT-26): It was created by Karner et al in 1979. The purpose of this questionnaire is to evaluate the symptoms of eating disorders and pathological eating attitudes and behaviors and to identify anorexia nervosa and anorexia nervosa. This questionnaire has 26 questions and 3 components including food habitation, hunger or desire to eat, and oral control. The lowest score awarded to a person is zero and the highest score is 78. If the person’s score in the test is higher than 21, they should be referred for further investigation and possibly treatment (of course, it does not necessarily indicate anorexia nervosa.^[20]^ Nobakht and Dejkam investigated the validity and reliability of EAT in Iranian population and found a correlation between the scores obtained from the 2 stages of the EAT questionnaire at 0.91, which indicates good reliability. The validity of the content of this questionnaire was also confirmed based on the opinion of experts in the research of Nobakht and Dejkam.^[21]^

- Body Image Shame Questionnaire (BISS): It was designed by Duarte et al in 2014 to measure a part of social shame that is related to a person’s body image. The final form of this tool has 14 items that are scored on a 5-point Likert scale from 0 equals never to 4 equals almost always. The questions 1 to 7 measure external shame and questions 8 to 14 measure internal shame. Higher scores shows higher level of shame. To check the internal consistency of this questionnaire, Duarte and colleagues used Cronbach alpha coefficient, the values of which were obtained for the whole test and the 2 subscales, respectively, 0.92, 0.08, and 0.90. Also, the retest coefficient with a 1-month interval has been calculated for the total score of the test and the scores for the 2 subscales, respectively, 75.06, 0.6, and 0.73.^[22]^ Sadeghzadeh et al examined the validity and reliability of the BISS questionnaire among Iranian male and female students. To assess internal consistency for the entire questionnaire and its 2 subscales, they used Cronbach alpha coefficient. The alpha values for the total scale and the external and internal shame subscales for female students were 0.91, 0.89, and 0.87, respectively. For the male student sample, these values were 0.90, 0.87, and 0.85, respectively. Furthermore, to evaluate temporal stability, test–retest reliability was conducted with a 2-week interval on a sample of 31 female students and 25 male students. The reliability coefficient for the total scale, external shame subscale, and internal shame subscale in the female student sample was 0.73, 0.71, and 0.66, respectively. For the male student sample, the reliability coefficients were 0.75, 0.73, and 0.69, respectively.^[23]^

In the present study, for descriptive analysis, quantitative data were presented using mean and SD, while qualitative data were presented using frequency (N) and percentage (%). Structural equation modeling was used to investigate the relationships between the studied variables (items).

In the Structural equation modeling, we can fit the goodness indices (Chi-square, Root Mean Square Error of Approximation (RMSEA), Comparative Fit Index (CFI), Relative Chi-square (χ²/df Ratio)). If (χ² (*P* > .05), RMSEA < 0.05, CFI ≥ 0.95, χ²/df Ratio: A ratio <3), the model has a good fit on the data.

SPSS software version 28 was used for the descriptive analysis of the data, to examine the relationships between the studied variables, and to measure and evaluate the accuracy of the AMOS model. The significance level for hypothesis testing was set at *P* < .05.

## 5. Results

In this study, 201 medical students with an average age of 22.2 ± 4.12 years participated. Additionally, the average BMI of the students was 22.72 ± 3.66. The majority of students (74.1%) did not have a history of mental or physical illness. Furthermore, most parents (39.3%) had bachelor degrees. Moreover, the majority of students (64.2%) had an average economic status, with only 1 student having an excellent economic status. The majority of students (60.2%) were undergraduate students (Table 1).

**Table 1:** Demographic characteristics of the participants.

Variable		N (%)
History of mental and physical illness	No	149 (74.1)
	Yes	52 (25.9)
Parents’ education	Diploma	70 (34.9)
	Bachelor’s	79 (39.3)
	General Doctorate	6 (3)
	Master’s	33 (16.4)
	Doctorate	13 (6.5)
Marital status	Single	188 (93.5)
	Married	13 (6.5)
Economic status	Very Poor	2 (1)
	Poor	11 (5.5)
	Average	129 (64.2)
	Good	58 (28.9)
	Excellent	1 (0.5)
Dormitory residence	No	65 (32.3)
	Yes	136 (67.7)
Field of study	Nursing	31 (15.4)
	Rehabilitation	17 (8.5)
	Other Para-medical Fields	44 (21.9)
	Midwifery	12 (6)
	Laboratory Sciences	11 (5.5)
	General Medicine	32 (15.9)
	Dentistry	42 (20.9)
	Pharmacy	12 (6)
Educational status	Bachelor’s	131 (65.2)
	Master’s	15 (7.5)
	General Doctorate (G.P)	46 (22.9)
	Specialized Doctorate (Ph.D)	9 (4.5)
Age (mean ± SD)	22.2±4.12	
Height (mean ± SD)	163.98±6.32	
Weight (mean ± SD)	61.17±11.01	
Body mass index (mean ± SD)	22.72±3.66	

The mean social media usage was reported as 38.79 ± 9.67, the mean body image shame was 17.77 ± 12.43, and the mean nutritional attitude was 38.42 ± 17.35 (Table 2).

**Table 2:** Frequency distributions of the items and subitems studied.

Items studied	Mean ± standard deviation (SD)
Social media usage	38.79 ± 9.67
Frequency of use	2.27 ± 1.07
Type of use	16.35 ± 4.45
Trust level with users	10.34 ± 4.2
Body Image Shame Scale (BISS)	17.77 ± 12.43
External Shame	1.7 ± 3.6
Internal Shame	10.05 ± 6.55
Eating Attitude Test-26 (EAT-26)	38.42 ± 17.35
Dietary habits	21.06 ± 11.91
Desire to eat	6.11 ± 4.48
Oral control	11.24 ± 5.66

Based on the obtained results, there was a significant relationship between BMI and body image shame (*P* < .05). This means that for every 1-unit increase in BMI, the body shame score increases by approximately 1 unit (0.96) (Table 3).

**Table 3:** Examination of the relationship between body image shame and body mass index, nutritional attitude and body mass index with univariate linear regression.

P-value	Coefficient	Variable
.004	0.96	Body image shame
<.001	1.02	Nutritional attitude

Also, there was a significant relationship between BMI and nutritional attitude (*P* < .05). This means that for every 1-unit increase in BMI, the nutritional attitude score increases by 1.02 (Table 3).

**Figure 1.**
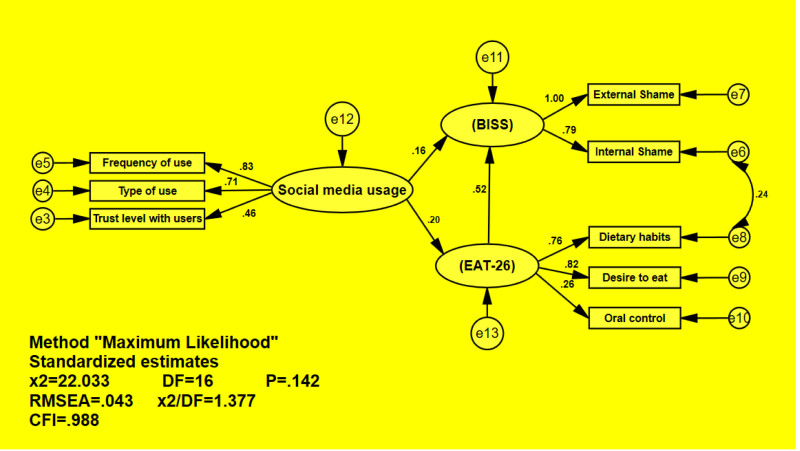
The confirmatory model of study.

In the current study, after validating the questionnaires, modeling, fitting, and predicting the variance of life satisfaction using the path analysis method and the AMOS software were investigated. The confirmatory model of this study is shown in Figure 1. Additionally, the estimates of the factor loads of the model are reported in Table 4.

**Table 4:** Estimate of factor loadings in the confirmatory model.

Path			Estimate	*P*
Social media usage	--->	Attitude towards nutrition	0.20	.034
Social media usage	--->	Body image shame	0.16	.034
Attitude towards nutrition	--->	Body image shame	0.52	<.001

Based on the reported results, the indicator of social media usage had a significant and positive direct effect on the attitude towards nutrition of students (*P* = .034). This means that as the score of social media usage increases, the score of students’ attitude towards nutrition also increases, such that for each 1-unit increase in the indicator of social media usage, the score of attitude towards nutrition increases by 0.20. Additionally, social media usage had a significant and positive direct effect on the indicator of body image shame of students (*P* = .034). As per your description, it appears that in the study, the higher the score of social media usage, the higher the score of body image shame of students, with an increase of 0.16 for each 1-unit increase in the social media usage indicator. Furthermore, students’ attitude towards nutrition had a significant and positive effect on the indicator of body image shame of students (*P* < .001). This means that the higher the score of students’ attitude towards nutrition, the higher the score of body image shame of students, with an increase of 0.52 for each 1-unit increase in the attitude towards nutrition indicator. Additionally, the results suggest a significant mediating effect of attitude towards nutrition on body image shame (*P* < .001). This means that social media usage has an indirect effect on body image shame through attitude towards nutrition, in addition to its direct effect.

Finally, to assess the model fit, appropriate indices were calculated and reported in Figure 1. Therefore, the Chi-square test assesses the discrepancy between the observed covariance matrix and the model-implied covariance matrix. It tests the null hypothesis that the model fits the data perfectly (*P* > .05), CFI indices compare the fit of the proposed model to an independent model (which assumes no relationship between the data) (CFI ≥ 0.95: Indicates a good fit), χ²/df Ratio (A ratio <3 is often considered acceptable). The RMSEA index, which measures the average residual correlations/covariances between the observed sample and the expected model from the population, is <0.08, indicating a good fit of the model.

## 6. Discussion

The purpose of this study was to examine the correlation between social media usage and the attitudes towards nutrition and body image shame among female students at Tehran University of Medical Sciences.

According to the results of the present study, the use of social media has a direct, positive, and significant effect on students’ body shame. The link between social media and body image dissatisfaction is complex and multifaceted. Research indicates that social media platforms, by allowing users to present curated and often heavily edited versions of their lives, contribute to the construction of unrealistic body standards. For instance, photo editing apps and filters can enhance appearance, creating an idealized version of oneself that does not correspond to reality.^[24]^ This discrepancy can lead to increased body dissatisfaction among viewers, as they compare their unedited selves to these idealized images.^[25]^ Moreover, social media often emphasizes visual content, where the appearance is given more importance than other attributes. This visual-centric nature can amplify preexisting societal pressures regarding body image. Studies have shown that engagement with appearance-focused social media content, such as fitspiration posts, is linked to greater body dissatisfaction and a higher likelihood of engaging in unhealthy weight control behaviors.^[26]^

Given the increasing use of social media in society, the level of influence from these media is continuously rising. This finding is consistent with the results of other studies, such as the study by Chukwuere et al (2021). This study employed a quantitative research methodology and aimed to explore the impact of social media platforms on students. This study used 201 students in first-year of university from the North-West University and determine different and widespread forms of social media platforms that students use daily, like Whatsapp, Facebook, Instagram, Twitter, Youtube, Tiktok, and Google. Its results indicated that social media platforms influence human life and social interactions. The positive and negative effects of social media on the interaction and social activities of students in the university environment and in society at large were reported to be significant.^[27]^

The present study found that nutritional attitudes significantly mediate the relationship between social media usage and body image shame. This suggests that the content consumed on social media can alter beliefs and behaviors related to food, which in turn impacts body image. Previous studies have highlighted the role of social media in promoting unhealthy dietary habits and distorted nutritional attitudes, especially among young women^[15,28]^ Interaction with social media and exposure to various visual content may negatively impact body image and food choices in young population groups, which are considered vulnerable. On the other hand, viewing images of beautiful celebrities, peers, fitness, and fashion or engaging in appearance comparisons increases the extent of these negative impacts.^[15]^ In the study by Ayyildiz and colleagues, the sample comprised 1411 university students. Data were collected through an online questionnaire, utilizing the Scale of Social Media Usage Motives, the Social Media Addiction Scale-Student Form, the Dutch Eating Behavior Questionnaire (DEBQ), the SCOFF Eating Disorders Scale, and the Life Assessment Scale. social media addiction among students was reported, and this addiction had a direct correlation with BMI, eating behavior, and life satisfaction.^[29]^ Notably, the results of some studies indicate that individuals with higher BMI are more prone to self-hatred and self-criticism, significantly displaying symptoms of anxiety and depression.^[30]^ Additionally, there is a high prevalence of body image dissatisfaction and body shame among students, affecting approximately 68% of women.^[31]^ The above results are consistent with the findings of this study, which demonstrated a significant correlation between BMI and body image shame.

The study by Lin et al (2020) and the participants in this study were high school students from Qazvin, a city in Iran. The inclusion criteria in this study were ages 13 to 18 years and a standardized BMI (z-BMI) indicating overweight or higher status with 2 gender. The result revealed a connection between eating disorders, food addiction, overweight, and psychological distress and these findings may provide healthcare providers with valuable insights to support the development of effective programs aimed at preventing excess weight issues among adolescents.^[32]^ Additionally, the present study reported a correlation between BMI and dietary attitudes. In a study conducted in 2021 on 398 Bangladeshi students, a significant relationship was observed between students’ weight and their dietary attitudes and behaviors. Specifically, as BMI increased,^[33]^ the dietary attitude score among these students also increased. Furthermore, a study conducted in Iran in the year 1400 aimed to examine and evaluate the relationship between attitudes and societal pressures on body image and eating disorders. The results of these studies align with the findings of the current study.^[34]^ Social media platforms often propagate dietary trends and body ideals that can contribute to unhealthy eating behaviors and distorted nutritional attitudes. For example, trends like clean eating, keto diets, and intermittent fasting are frequently promoted by influencers, often without a basis in scientific evidence.^[35]^ These trends can lead to restrictive eating behaviors, which are associated with increased body dissatisfaction and higher risk for developing eating disorders.^[36]^ Additionally, the phenomenon of “fitspiration,” which combines fitness and inspiration, has been shown to have both positive and negative effects. While some individuals may feel motivated to adopt healthier lifestyles, others may experience increased body dissatisfaction and disordered eating behaviors due to the often unrealistic and highly idealized portrayals of fitness and health.^[37]^ The pressure to achieve a fit and toned body, as presented by social media influencers, can lead to extreme dietary restrictions and compulsive exercise behaviors, which are detrimental to mental and physical health.^[36]^

The findings of present study have important implications for interventions aimed at reducing body dissatisfaction and promoting healthy nutritional attitudes among young women. Given the significant impact of social media, there is a need for programs that educate young women about the unrealistic nature of the images they see online and promote critical thinking about the content they consume.^[38]^ Additionally, interventions should focus on promoting body positivity and self-acceptance, as well as providing support for those struggling with body image issues and disordered eating behaviors.

## 7. Limitations and future research

Despite the valuable insights gained from this study, there are several limitations that should be acknowledged. First, the cross-sectional design limits the ability to draw causal inferences; longitudinal studies are needed to establish the directionality of the relationships observed. Second, the study relied on self-reported measures, which may be subject to recall bias, leading to inaccuracies in self-perception. Third, the analysis should either account for additional confounders such as socioeconomic status, mental health history, or physical activity levels, which may influence the relationships studied.

Additionally, since the study focused on female students at Tehran University of Medical Sciences and used convenience sampling, caution is needed when generalizing the results to the broader population. Future research should explore these relationships in more diverse populations, including males and individuals from different cultural backgrounds. It would also be valuable to examine the role of specific social media platforms and types of content (e.g., fitness influencers, beauty tutorials) in shaping nutritional attitudes and body image. Additionally, future studies should consider other potential confounding variables, such as socioeconomic status, mental health history, and physical activity levels, which may influence the relationships under investigation.

## 8. Conclusion

In conclusion, this study highlights the significant impact of social media usage on nutritional attitudes and body image shame among female students at Tehran University of Medical Sciences. The findings underscore the need for interventions that address the negative effects of social media on young women’s perceptions of their bodies and their relationship with food. By promoting media literacy, body positivity, and healthy nutritional attitudes, it is possible to mitigate the harmful effects of social media and support the well-being of young women.

## Acknowledgments

The researchers express their gratitude to the Research Vice Presidency of Tehran University of Medical Sciences and the participants of the study.

## Author contributions

**Conceptualization:** Sogol Keyvanpour, Masoumeh Namazi.

**Data curation:** Narges Ebrahimi.

**Investigation:** Sogol Keyvanpour, Narges Ebrahimi.

**Methodology:** Fatemeh Hosseini.

**Software:** Fatemeh Hosseini.

**Supervision:** Masoumeh Namazi.

**Validation:** Fatemeh Hosseini.

**Visualization:** Narges Ebrahimi.

**Writing – original draft:** Sogol Keyvanpour, Masoumeh Namazi.

**Writing – review & editing:** Masoumeh Namazi.
